# Cross-cultural adaptation and patients' judgments of a Question Prompt List for Italian-speaking cancer patients

**DOI:** 10.1186/1472-6963-10-16

**Published:** 2010-01-15

**Authors:** Caterina Caminiti, Francesca Diodati, Silvia Filiberti, Barbara Marcomini, Maria Antonietta Annunziata, Maria Ollari, Rodolfo Passalacqua

**Affiliations:** 1 Research & Innovation Unit, University Hospital of Parma, Italy; 2 Division of Medical Oncology, Istituti Ospitalieri, Cremona, Italy; 3 Unit of Oncological Psychology, Istituto Nazionale Tumori, Aviano, Italy

## Abstract

**Background:**

Question Prompt Lists (QPLs) have proven very effective in encouraging cancer patients to ask questions, allowing them to take up a more active role during visits with the oncologist. As no such tool has yet been validated for Italian-speaking users, we carried out the cross-cultural adaptation and evaluation of an existing Australian Question Prompt List.

**Methods:**

Cross-cultural adaptation was performed in accordance with the five steps described by Guillemin and Beaton. Forward and back translations of the original tool were carried out, and the products discussed by an Expert Committee who agreed on a prefinal version of the Italian QPL, which was submitted to 30 volunteer patients for evaluation. They rated each question's adequacy of content, clarity of wording, usefulness, and generated anxiety, on a 3-point Likert scale. Based on the analysis of patient ratings, the final version of the Italian QPL was produced.

**Results:**

Few discrepancies between the two back translations and the original version of the instrument were noted, indicating that the Italian translation (synthesis of the 2 forward translations) was substantially accurate. Most volunteer patients felt that the questionnaire was adequate, easy to understand and useful. Only a few minor criticisms were expressed. Certain questions on diagnosis and prognosis generated the highest level of anxiety. Patient comments and ratings on clarity highlighted the need to clarify common health care terms which are not widely used by the public (i.e. guideline, multidisciplinary team and clinical trial)

**Conclusions:**

This cross-cultural adaptation has produced an Italian Question Prompt List that is now available for multi-center international studies and can be safely used with Italian-speaking cancer patients.

## Background

Compared to technical aspects of patient care, communication between the patient and health care professionals has often been considered an issue of minor importance, rarely included in medical curricula [1]. In reality, communication skills are the cornerstone of comprehensive cancer care. It is increasingly accepted that good communication is a key to achieving important goals of the clinical encounter in oncology: the quality of the oncologist-patient communication has been shown to affect crucial aspects including satisfaction with care, decision making, accrual to clinical trials, pain and other symptoms, and patient distress [1,2]. Numerous studies have shown that the vast majority of cancer patients wishes to have more information concerning diagnosis, prognosis and therapeutic options, as well as a better dialogue with clinicians, even in Italy, where a generally non-disclosure culture was thought to predominate [3-6]. Despite these attitudes, these necessities often remain unheeded. On the one hand, medical oncologists and healthcare staff generally underestimate this need, find it difficult to reveal prognostic uncertainties, and underestimate patient knowledge of medical terms [7-9]; furthermore, particularly in countries with a tradition of non-disclosure like Italy, clinicians tend to withhold information, believing this is what patients want and need [10,11]. In Italy this situation has led to the establishment of information campaigns devoted to alternative forms of therapy that have caused serious harm to patients and public health care services as a whole [10].

On the other hand, patients themselves are often reluctant or unable to initiate conversations on issues of concern, for various reasons: they are afraid they might be annoying or taking up too much of a busy doctor's time, they fear their illness and the answers they may receive, they are afraid of sounding ignorant, or are confident that the physician will tell them all they need to know [1,12]. Based on these considerations, tools aiming to favor patient-physician communication and to encourage patient participation in the medical consultation and decision-making process are urgently needed.

Question Prompt Lists (QPLs), consisting of a structured list of questions that patients may wish to ask their physician, have been shown to facilitate communication between patients and physicians about illness and treatment, as measured objectively through the number of patient questions asked or subjectively through patient self-report [13]. QPLs are generally given patients before the consultation, leaving them enough time to read through them and mark the questions that they would like to ask.

To our knowledge, no research on the use of QPLs in Italy has been reported. Given the positive results obtained with this tool in English-speaking countries, it is essential that this tool be offered to cancer patients from other countries and cultures as well.

This study was sponsored by the Italian Ministry of Health and by the Lombardy Region, as part of a broader implementation project aiming to introduce in Italian cancer centers a series of evidence-based activities for the improvement of the humanization of cancer care (HUCARE project). Such activities include communication courses for medical oncologists and nurses, the construction of a PIS (Point of Information and Support) inside the hospital ward [14], the assignment of a referring nurse to all new patients and the measuring of psychological distress for all cancer patients. The adoption of a QPL is one of the activities of the HUCARE project for the improvement of doctor-patient communication, which obtained approval from the Ethics Committee of Cremona. Instead of devising and validating our own Question Prompt List, given the availability of already validated, widely used QPLs in the English language, we decided to carry out the cross-cultural adaptation of one of these existing instruments, and to test it on a selected group of Italian cancer patients.

## Methods

### The Instrument

Literature analysis was carried out to identify reports of Question Prompt List use in oncology. We only found experiences relating to English-speaking countries, with no report of cross-cultural adaptation in any language. A metanalysis of such studies, conducted at the Medical Psychology Research Unit (MPRU) at the University of Sydney, Australia, shows that more than half of such studies were carried out at the MPRU itself [13]. Given the extensive research on the subject by this Australian group, we opted to employ the instrument devised at the MPRU, presented in the above mentioned metanalysis by Dimoska et al, dedicated to the communication with the medical or radiation oncologist, available for download from the MPRU website [15].

The booklet contains 49 questions, subdivided into eleven domains: "how and when to ask questions", "diagnosis", "tests", "prognosis", "optimal care", "the multidisciplinary team", "treatment information and options", "clinical trials", "preparing for treatment", "costs", "support information".

Permission to translate and adapt the tool was obtained from the developers.

### Cross-cultural adaptation

Translating a tool into another language is not enough to ensure validity. Cross-cultural adaptation is a complex, well-defined process, aiming to ensure that the translated version is culturally sensitive, as large variations exist between cultures in attitudes toward disease, participation in decision-making, and in discussing illness [13]. Guidelines have been developed delineating the necessary steps for this task, however no such guideline exists on the cross-cultural adaptation of a QPL [16]. We thus followed the five steps described by Guillemin et al [17] and Beaton et al [18], intended for questionnaires of self-report health status measures, as follows.

Stage I - initial translation: two certified translators with Italian as their mother tongue (Translator1 and Translator2) independently translated the tool into Italian producing two forward translations defined as T1 and T2. As recommended by Beaton et al. [18], the translators had different backgrounds: Translator1, one of the co-authors of this paper, is employed at the University Hospital of Parma, and is a member of the HUCARE Project's Coordination group, thus fully aware of the study's rationale and aims, while Translator2 is a certified translator with no specific background in oncology, and was not informed of the aims of the study nor of the existence of the HUCARE project.

Stage II - synthesis: The two translators met to discuss their work, and agreed on a common Italian version, defined as T12. A recording observer made note of discrepancies between the translations and difficulties encountered.

Stage III - back translation: two English native speaking certified translators translated T12 into English. Both had no specific background in oncology, nor were they informed of the aims of the study and of the existence of the Hucare Project.

Stage IV - Expert Committee - a Committee was set up with the aim to examine all translated versions against the original tool, and produce the so-called "prefinal version" of the QPL. The composition of the Committee, in strict accordance with the requirements of Beaton et al [18] included the following figures: the four translators, one oncologist - also the project manager -, one methodologist-biostatistician, one psychologist, one sociologist, one linguist. Each question was discussed and evaluated from all the viewpoints of the different professionals in the Committee, particularly concerning semantic equivalence, idiomatic equivalence, experiential equivalence and contextual equivalence.

Stage V - test of the prefinal version: the Italian version agreed on by the Committee was shared with a group of oncologists, the Directors of 7 of the cancer centers participating in the HUCARE project, to obtain endorsement, and was subsequently submitted to a sample of cancer survivors (former patients or currently in follow-up), recruited from two voluntary patient organizations based in the Northern Italian towns of Reggio-Emilia and Cremona. A psychologist presented the QPL and its objectives to the patients, and requested them to rate each question's adequacy of content (if the wording and content of the question was adequate to the context), clarity of wording, usefulness, and the level of anxiety it generated, using a 3-point Likert scale, as well as to provide any comments or suggestions.

In line with the recommendations of the International Society for Pharmacoeconomics and Outcomes Research (ISPOR) Task Force for Translation and Cultural Adaptation [16], the prefinal translation was reviewed in the light of the findings of Stage V, proofread for any spelling or grammatical errors, and finalized with the approval of the project manager.

### Statistical analysis

**Figure 1:**
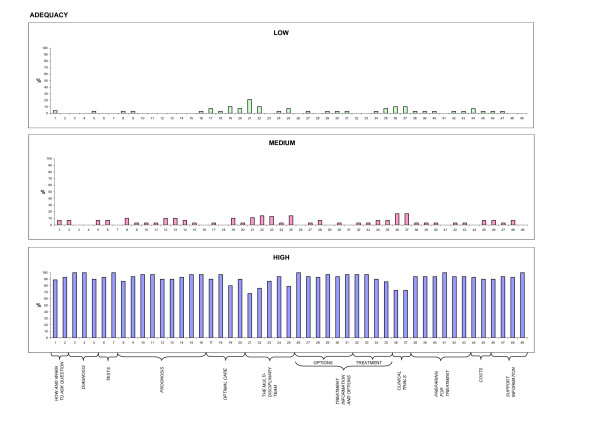
**Adequacy**. Patient ratings on adequacy of content of each question, expressed on a 3-point Likert scale.

**Figure 2:**
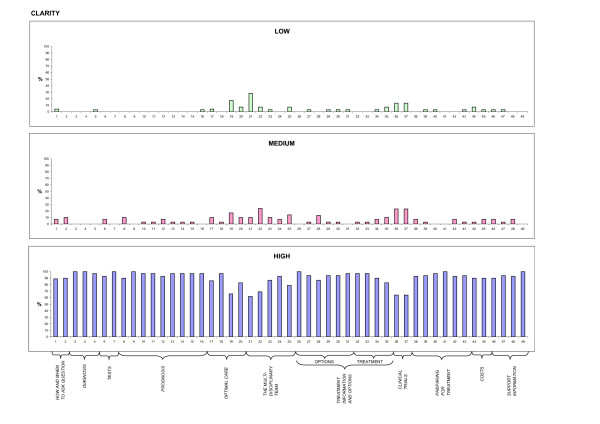
**Clarity**. Patient ratings on clarity of wording of each question, expressed on a 3-point Likert scale.

**Figure 3:**
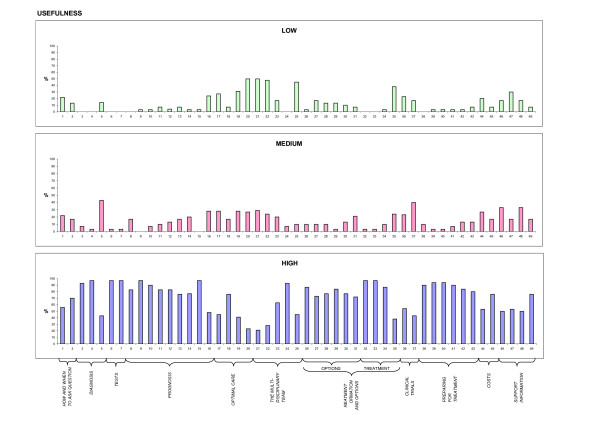
**Usefulness**. Patient ratings on usefulness of each question, expressed on a 3-point Likert scale.

**Figure 4:**
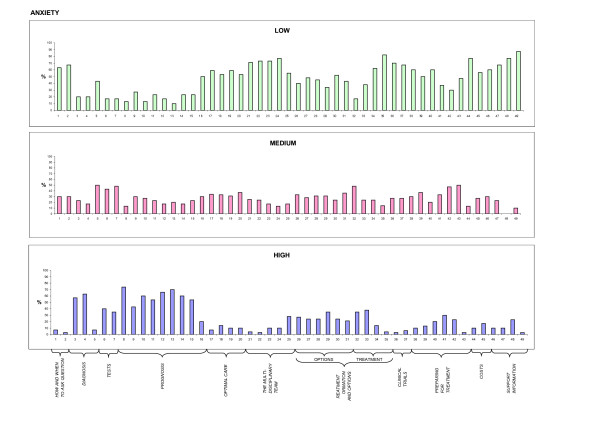
**Anxiety**. Patient ratings on anxiety generated by each question, expressed on a 3-point Likert scale.

Descriptive analysis of patient feedback (stage V) was carried out, considering the scores attributed to each item of the QPL. To facilitate comprehension, and highlight differences, frequencies were displayed by means of histograms (Figures 1, 2, 3, and 4). 50% cut offs were considered as significant for analysis by the Committee.

## Results

A total of 49 questions underwent cross-cultural adaptation (see additional file 1: QPL final version following patient evaluation). All five stages were carried out in strict adherence to the indications of the literature [18]. Concerning Stage II and III of the process, few discrepancies between the two back translations and the original version of the instrument were noted, indicating that T12 (synthesis of the 2 forward translations) was substantially accurate.

In stage IV, the directness of the language used in the Australian QPL, typical of English-speaking cultures, proved to be the major source of debate among the Committee. In some cases, items were slightly rephrased to make them less direct, still ensuring to maintain the original meaning. A faithful translation of the expression "so you have cancer..." was unanimously considered unacceptable for Italian users, and therefore was removed from the Italian version. The Committee also agreed not to use the Italian counterpart for "cancer", which carries a strong connotation of malignancy, although some Italian experts now feel the word should be used with patients. The milder term "tumor" was preferred, or in some cases, "cancer" was translated with the phrase "my illness", as this expression is often used by Italian-speaking oncology patients referring to their disease. In other instances, terms judged to be particularly harsh were maintained, as not to impact on the meaning, e.g. the expression "cancer survivor", which may sound somewhat disturbing to some Italian patients.

Other points of discussion within the Committee concerned the choice of technical vs. more colloquial terms. Generally, it was decided to keep the language informal and simple, following the English version, although in some cases the wording was slightly modified to better suit the Italian style.

A few idiomatic expressions proved problematic: in particular, the heading "Optimal care" was challenging for the forward translators, consequently causing the back translations of the phrase to be incongruent with the original version. The Committee finally agreed on a somewhat free translation of the heading: "Ensuring the best possible care".

The original QPL contained a few English words that are also used in Italian "checklist, follow-up, team". The Committee however chose to replace them with Italian terms, as they may not be known by all possible users.

From a grammatical point of view, the Committee took care to leave open questions open, although in some instances a closed question would have sounded better to Italian ears.

30 patients participated in the evaluation of the items, completing Stage V of the cross-cultural adaptation process. 60% of the sample was female, median age was 60 years; level of education was as follows: 13% primary school, 20% secondary school, 60% highschool, and 7% University graduate. Patient ratings of each item concerning the four dimensions of adequacy of content, clarity of wording, usefulness, and level of anxiety, expressed on a 3-point Likert scale, are depicted in Figures 1, 2, 3, and 4, respectively. For each dimension, three histograms were constructed, relative to the three scores: low, medium, and high. In each histogram, the X-axis depicts the 49 questions, and the Y-axis shows the frequency of the score expressed as percentages. To facilitate interpretation, the eleven domains, into which the questions were subdivided in the booklet, are highlighted.

Most questions received high scores (> 90%) for adequacy of content and clarity of wording (Figures 1 and 2). Ratings for clarity were slightly lower (< 70%) only for questions 19, 21, 22, 36, and 37 (see additional file 1: QPL final version following patient evaluation). Concerning usefulness, results were less uniform (Figure 3). Scores were particularly high for most questions in the "prognosis", "treatment information and options" and "preparing for treatment" domains. A few items received low scores: questions 20, 21, 22, 25, 35 (see additional file 1: QPL final version following patient evaluation). What these questions have in common is that they investigate factors external to the patient-physician relationship and not referring to the patient condition (second opinion, the multidisciplinary team, public vs. private institutions).

The level of anxiety generated by questions was judged low for most items (Figure 4). Only for the domains of diagnosis and prognosis, most questions received high scores (> 50% of patients).

The evaluation sheet also provided space next to each item where respondents could leave comments, which would be useful to identify frequent problems or observations. Comments were provided by 9 out of 30 subjects, and were most frequent in the "Optimal Care" and "The Multidisciplinary Team" domains. In particular, 5 patients indicated that they did not know the meaning of the terms "guidelines" and "multidisciplinary team".

Based on the abovementioned findings, the Committee decided to revise the prefinal version, rephrasing questions 19, 21, 22, 36 and 37 in order to improve clarity.

Questions 20 and 25, regarding seeking a second opinion and the relationship to multiple members of a team, were not removed, despite their low usefulness scores, as the group considered these to be crucial issues requiring special attention in the Italian culture. Similarly, question 35, concerning care in the public vs. private sectors, was not deleted despite its low usefulness scores. In fact, although differences between public and private institutions are not so evident in Italy, the question would be relevant to Italian speaking patients living in other countries.

## Discussion

The evidence supporting the positive effects of QPLs in oncology obtained in English-speaking countries call for an extension of their availability to other linguistic groups. To our knowledge, this is the first report of the cross-cultural adaptation of a QPL for cancer patients.

By strictly following the indications in the literature for cross-cultural adaptation of an instrument, we have ensured that the translated questionnaire is suitable for use with Italian-speaking cancer patients.

The multidisciplinary nature of the Expert Committee was crucial in reaching an appropriate version, examining the work from different points of view: oncologist, methodologist, translator, linguist, psychologist, sociologist. The major obstacles encountered by the Committee seemed to lie in the directness of the language used in the Australian QPLs, which required some degree of "mitigation" of the language, obviously without modifying the meaning of the items.

Finally, judgments expressed by patients on each question in stage V of the process, provided the indispensable patient perspective to ensure that the tool may be used safely with newly diagnosed cancer patients, who are certainly under considerable stress and much more vulnerable. Patient feedback provided an insight into the Italian culture, confirming that the somewhat paternalistic nature of the patient-physician relationship is still present. The fact that many respondents did not consider useful items relating to seeking a second opinion and handling instructions from health care professionals other than their oncologist may suggest a lack of patient empowerment, and a tendency to entrust care decisions to the doctor. The Committee decided to maintain these items despite their low usefulness ratings, since the aim of the QPL is actually to favor patient empowerment and fulfill patient information needs, which may not even be always realized fully by the patients themselves. The literature supports this view: in a 1999 survey conducted on 1120 Italian cancer patients, more than half of respondents (53%) declared that they would base their choice of treatment on their doctor's recommendations, and only 32% on scientific evidence [3].

The feedback obtained by the patients in our survey is in line with findings of research on QPL use. In particular, English QPLs had been shown to increase likelihood that a patient would ask at least 1 question about prognosis [19], a topic that is typically avoided by both cancer patients and physicians during the consultation [20]. In fact, the "prognosis" domain was ranked among the first for usefulness by our sample.

Scores for anxiety generated by questions were high for many items in the "diagnosis" and "prognosis" domains. This was expected, considering the impact of a cancer diagnosis and its prognosis on a person's life. However, there is evidence that lack of information can actually increase anxiety and uncertainty, while good communication was reported to be associated with better emotional adjustment [11]; findings which are part of the rationale for QPL introduction in oncology settings. Contrary to what feared by some clinicians, the review by Dimoska et al reports that no study detected a negative effect of the QPL on psychological outcomes, and that 2 papers demonstrated anxiety actually decreased after the patient received the QPL.

This initiative is part of a large, multicenter, nationwide implementation study financed by the Italian Ministry of Health, the HUCARE Project, aiming to introduce evidence-based interventions to cancer centers with the objective of "humanizing" the care of cancer patients by improving patient information, patient-physician communication, as well as detection and fulfillment of patient psychosocial needs. The introduction of a QPL in all participating centers is one of the key interventions foreseen in the Hucare protocol, which contains specific indications on its use, in strict accordance with published evidence. As indicated by the Australian research group [13], the QPL will be given to patients by their oncologist, who will explain to them its content and purpose, reassuring them that asking questions is their right and reiterating his/her willingness to answer any concern, no matter how trivial it may seem to the patient. The Hucare project also includes specific training for physicians on patient-clinician communication, where all aspects of QPL use are discussed.

## Conclusions

Enhancing communication between patients and physicians is a key aspect of cancer care. The process of cross-cultural adaptation we followed has produced a Question Prompt List that is now available for multi-center international studies and can be safely used with Italian-speaking cancer patients. All self-administered instruments aimed at the improvement of care (health assessment questionnaires, questionnaires on patient needs, depression and anxiety scales, etc.) should be translated and validated in order to be used by all patients, regardless of their culture and language.

## Competing interests

The authors declare that they have no competing interests.

## Authors' contributions

CC conceived of the study, designed the methodology, performed data interpretation and supervised the entire process, up to the drafting of the manuscript. FD was one of the two forward translators, performed literature review and contributed to the drafting of the manuscript. SF was in charge of stage V of the process, she recruited patients and assisted them in the completion of the self-administered questionnaire. BM carried out data analysis and developed the graphs used for result interpretation. MAA and MO contributed to the work of the Expert Committee. RP is the coordinator of the Hucare project and devised all interventions foreseen in the Hucare protocol, including QPL introduction; he was responsible for the entire process: selection of all figures involved, coordination of meetings, and decision-making based on the study results.

All authors read and approved the final manuscript.

## Pre-publication history

The pre-publication history for this paper can be accessed here:

http://www.biomedcentral.com/1472-6963/10/16/prepub

## Supplementary Material

Additional file 1 **QPL final version following patient evaluation**. The 49 original questions and the Italian final version, grouped into the 11 domains. Click here for file

## References

[B1] Fallowfield L Jenkins V Effective communication skills are the key to good cancer care Eur J Cancer 1999 35 1592 1597 10.1016/S0959-8049(99)00212-9 10673967

[B2] Baile WF Aaron J Patient-physician communication in oncology: past, present, and future Curr Opin Oncol 2005 17 331 335 10.1097/01.cco.0000167738.49325.2c 15933462

[B3] Passalacqua R Campione F Caminiti C Salvagni S Barilli A Bella M Barni S Barsanti G Caffo O Carlini P Cinquemani G Di Costanzo F Giustini L Labianca R Mazzei A Olmeo N Paccagnella A Toscano L Cocconi G Patients' opinions, feelings, and attitudes after a campaign to promote the Di Bella therapy Lancet 1999 353 1310 1314 10.1016/S0140-6736(98)10253-2 10218529

[B4] Jenkins V Fallowfield L Saul J Information needs of patients with cancer: results from a large study in UK cancer centres Br J Cancer 2001 84 48 51 10.1054/bjoc.2000.1573 11139312 PMC2363610

[B5] Yun YH Lee CG Kim SY Lee SW Heo DS Kim JS Lee KS Hong YS Lee JS You CH The attitudes of cancer patients and their families toward the disclosure of terminal illness J Clin Oncol 2004 22 307 314 10.1200/JCO.2004.07.053 14722040

[B6] Piredda M Rocci L Gualandi R Petitti T Vincenzi B De Marinis MG Survey on learning needs and preferred sources of information to meet these needs in Italian oncology patients receiving chemotherapy Eur J Oncol Nurs 2008 12 120 126 10.1016/j.ejon.2007.10.004 18294913

[B7] Chapman K Abraham C Jenkins V Fallowfield L Lay understanding of terms used in cancer consultations Psychooncology 2003 12 557 566 10.1002/pon.673 12923796

[B8] Mystakidou K Parpa E Tsilila E Katsouda E Vlahos L Cancer information disclosure in different cultural contexts Support Care Cancer 2004 12 147 154 10.1007/s00520-003-0552-7 15074312

[B9] Hagerty RG Butow PN Ellis PM Dimitry S Tattersall MH Communicating prognosis in cancer care: a systematic review of the literature Ann Oncol 2005 16 1005 1053 10.1093/annonc/mdi211 15939716

[B10] Passalacqua R Caminiti C Salvagni S Barni S Beretta GD Carlini P Contu A Di Costanzo F Toscano L Campione F Effects of media information on cancer patients' opinions, feelings, decision-making process and physician-patient communication Cancer 2004 100 1077 1084 10.1002/cncr.20050 14983505

[B11] Costantini M Morasso G Montella M Borgia P Cecioni R Beccaro M Sguazzotti E Bruzzi P ISDOC Study Group Diagnosis and prognosis disclosure among cancer patients. Results from an Italian mortality follow-back survey Ann Oncol 2006 17 853 859 10.1093/annonc/mdl028 16551764

[B12] Jefford M Tattersall MH Informing and involving cancer patients in their own care Lancet Oncol 2002 3 629 637 10.1016/S1470-2045(02)00877-X 12372725

[B13] Dimoska A Tattersall MH Butow PN Shepherd H Kinnersley P Can a "prompt list" empower cancer patients to ask relevant questions? Cancer 2008 113 225 237 10.1002/cncr.23543 18484592

[B14] Passalacqua R Caminiti C Campione F Diodati F Todeschini R Bisagni G Labianca R Dalla Chiesa M Bracci R Aragona M Artioli F Cavanna L Masina A De Falco F Marzocchini B Iacono C Contu A Di Costanzo F Bertetto O Annunziata MA Prospective, multicenter, randomized trial of a new organizational modality for providing information and support to cancer patients J Clin Oncol 2009 27 1794 1799 10.1200/JCO.2007.15.0615 19273715

[B15] MPRU - Medical Psychology Research Unit http://www.psych.usyd.edu.au/mpru/communication_tools.html (accessed August 2009)

[B16] Wild D Grove A Martin M Eremenco S McElroy S Verjee-Lorenz A Erikson P ISPOR Task Force for Translation and Cultural Adaptation Principles of Good Practice for the Translation and Cultural Adaptation Process for Patient-Reported Outcomes (PRO) Measures: report of the ISPOR Task Force for Translation and Cultural Adaptation Value Health 2005 8 94 104 10.1111/j.1524-4733.2005.04054.x 15804318

[B17] Guillemin F Bombardier C Beaton D Cross-cultural adaptation of health-related quality of life measures: literature review and proposed guidelines J Clin Epidemiol 1993 46 1417 1432 10.1016/0895-4356(93)90142-N 8263569

[B18] Beaton DE Bombardier C Guillemin F Ferraz MB Guidelines for the process of cross-cultural adaptation of self-report measures Spine (Phila Pa 1976) 2000 25 3186 3191 11124735 10.1097/00007632-200012150-00014

[B19] Brown R Butow P Dunn S Tattersall M Promoting patient participation and shortening cancer consultations: a randomised trial Br J Cancer 2001 85 1273 1282 10.1054/bjoc.2001.2073 11720460 PMC2375236

[B20] Tamburini M Gangeri L Brunelli C Boeri P Borreani C Bosisio M Karmann CF Greco M Miccinesi G Murru L Trimigno P Cancer patients' needs during hospitalisation: a quantitative and qualitative study BMC Cancer 2003 3 12 10.1186/1471-2407-3-12 12710890 PMC155542

