# Supplementary material for: Cross-cultural adaptation and patients' judgments of a Question Prompt List for Italian-speaking cancer patients
Source: BMC Health Serv Res. 2010 Jan 15;10:16. doi: 10.1186/1472-6963-10-16 (PMC2821383; doi:10.1186/1472-6963-10-16)
Supplement: Additional file 1 — QPL final version following patient evaluation. The 49 original questions and the Italian final version, grouped into the 11 domains. [file 1472-6963-10-16-S1.DOC]

**Appendix - Final version of the QPL following patient evaluation**

| **Nr. QUESTION** | **QUESTION FROM ORIGINAL BOOKLET** | **ITALIAN VERSION** |
| --- | --- | --- |
|  | HOW AND WHEN TO ASK QUESTIONS | COME E QUANDO CHIEDERE |
| 1 | Do you have time today to discuss my questions? | Oggi ha un po’ di tempo per rispondere alle mie domande? |
| 2 | Can I ask you to explain any words that I am not familiar with? | Posso chiederle di spiegarmi il significato di termini che non conosco bene? |
|  | DIAGNOSIS | DIAGNOSI |
| 3 | What kind of cancer do I have? | Che tipo di tumore ho? |
| 4 | Where is the cancer at the moment? Has it spread to other parts of my body? | Dove si trova il tumore adesso? Si è esteso ad altre parti del corpo? |
| 5 | How common is my cancer? | Quanto è comune la mia malattia? |
|  | TESTS | ESAMI |
| 6 | Are there any further tests that I need to have? What will they tell us? Will they confirm my diagnosis? | Devo fare altri esami? Quali altre informazioni ci daranno? Confermeranno la mia diagnosi? |
| 7 | What will I experience when having the test/s? | Cosa mi succederà durante questo/i esame/i? |
|  | PROGNOSIS | PROGNOSI |
| 8 | How bad is this cancer and what is it going to mean for me? | Quanto è grave questo tumore e cosa significherà per me? |
| 9 | What symptoms will the cancer cause? | Quali saranno i sintomi? |
| 10 | What is the aim of the treatment? To cure the cancer or to control it and manage symptoms? | Qual è l’obiettivo della terapia? Guarirmi dal tumore o tenerlo sotto controllo e alleviare i sintomi? |
| 11 | How likely is it that the cancer will spread to other parts of my body without any more treatment? | Senza ulteriori terapie, qual è la probabilità che il tumore si estenda ad altre parti del corpo? |
| 12 | How likely is it that the cancer will spread to other parts of my body if I do have more treatment? | Se decido di sottopormi ad ulteriori terapie, qual è la probabilità che il tumore si estenda ad altre parti del corpo? |
| 13 | What is the expected survival for people with my type of cancer? | Qual è l’aspettativa di vita per chi ha il mio stesso tumore? |
| 14 | Is the treatment going to improve my chance of survival? | La terapia migliorerà le mie probabilità di sopravvivenza? |
| 15 | How likely is it that the treatment will improve my symptoms? Is it worth going through? | Qual è la probabilità che la terapia migliori i miei sintomi? Vale la pena affrontarla? |
| 16 | Will the treatment or illness reduce my sexual drive? | E’ possibile che la terapia, o la malattia, riducano il mio desiderio sessuale? |
|  | OPTIMAL CARE | ASSICURARSI LE CURE MIGLIORI |
| 17 | Do you specialise in treating my type of cancer? | Lei è specializzato nel trattamento di un tumore come il mio? |
| 18 | How well established is the treatment you are recommending? | Quanto è utilizzata e consolidata la terapia che mi consiglia? |
| 19 | Are there guidelines on how to treat my cancer? | Ci sono linee guida per il trattamento della mia malattia, cioè documenti che contengono raccomandazioni su diagnosi e terapie di questo tipo di tumore, su cui concorda la maggior parte degli esperti? |
| 20 | Is there another specialist who treats this type of cancer that you recommend for a second opinion? | Conosce qualche altro specialista che tratta il mio tipo di tumore a cui potrei rivolgermi per una seconda opinione? |
|  | THE MULTI-DISCIPLINARY TEAM | L’EQUIPE MULTIDISCIPLINARE |
| 21 | Do you work in a multi-disciplinary team and what does this mean? | Lei lavora in una equipe multidisciplinare, cioè collabora con colleghi specializzati in diversi aspetti della mia malattia? |
| 22 | Can you explain the advantages of a team approach? | Può spiegarmi i vantaggi di un lavoro di equipe, cioè della collaborazione con colleghi specializzati nella mia malattia? |
| 23 | How do you all communicate with each other and me? | Come si svolgerà la comunicazione tra di voi e tra voi e me? |
| 24 | Who will be in charge of my care? | Chi mi seguirà nel percorso di cura? |
| 25 | What do I do if I get conflicting information? | Come mi devo comportare se mi vengono date informazioni contrastanti? |
|  | TREATMENT INFORMATION AND OPTIONS | INFORMAZIONI E OPZIONI TERAPEUTICHE |
|  | *Options* | *Opzioni* |
| 26 | Is it necessary to have treatment right now? | E’ proprio necessario cominciare la terapia ora? |
| 27 | If so, do I have a choice of treatments? | Se sì, posso scegliere tra diversi trattamenti? |
| 28 | What are the pros and cons of each treatment option? | Quali sono i pro e i contro di ciascuna opzione terapeutica? |
| 29 | What can I expect if I decide not to have treatment? | Cosa posso aspettarmi nel caso decidessi di non sottopormi ad alcun trattamento? |
| 30 | How much time do I have to think about this? Do you need my decision today? | Quanto tempo ho per pensarci? Devo decidere oggi stesso? |
| 31 | What is your opinion about the best treatment for me? | Secondo lei qual è la migliore terapia per il mio caso? |
|  | *Treatment* | *Terapia* |
| 32 | What exactly will be done during the treatment and how will it affect me? When are these effects likely to happen? | Come si svolgerà esattamente la terapia e che effetti avrà su di me? Presumibilmente quando si verificheranno questi effetti? |
| 33 | What is the treatment schedule, e.g. how many treatments will I have, how often, and for how long will I have treatment? | Qual è il programma terapeutico che dovrò seguire, es. quanti cicli di terapia, con che frequenza, e per quanto tempo? |
| 34 | Where will I have the treatment? | Dove farò la terapia? |
| 35 | Are there any dvantages/disadvantages of the private versus public health system? | Ci sono vantaggi/svantaggi tra strutture private e pubbliche? |
|  | CLINICAL TRIALS | STUDI CLINICI |
| 36 | What are clinical trials? Are there any that might be relevant for me? | Ci sono studi in corso che potrebbero applicarsi al mio caso? |
| 37 | Will I be treated any differently if I enrol in a trial? | Sarei curato diversamente se prendessi parte a uno studio? |
|  | PREPARING FOR TREATMENT | PREPARAZIONE ALLA TERAPIA |
| 38 | Is there anything that I can do before or after my treatment that might make it more effective, e.g. diet, work, exercise, etc? | C’è qualcosa che potrei fare prima o dopo la terapia per renderla più efficace,(alimentazione, lavoro, esercizio fisico, ecc.)? |
| 39 | What are the do’s and don’ts while having treatment? | Quali sono le cose che si possono o non si possono fare durante la terapia? |
| 40 | What problems should I look out for and who do I contact if they occur? | Che problemi potrebbero insorgere e chi dovrò contattare se si verificheranno? |
| 41 | Are there long-term side effects from the treatment? | La terapia comporta effetti collaterali a lungo termine? |
| 42 | Will I need any additional treatment after this? If so, what might that be? | Dovrò sottopormi ad altri trattamenti dopo questo? Se sì, quali? |
| 43 | What is my long-term follow up plan? | Che controlli dovrò fare dopo la fine della terapia? |
|  | COSTS | COSTI |
| 44 | What will be the costs throughout my treatment, e.g. medication, chemotherapy, etc? | Che spese dovrò sostenere durante tutto il trattamento, es. farmaci, chemioterapia, ecc.?) |
| 45 | Am I eligible for any benefits if I cannot work? | Avrò diritto a qualche indennità se non potrò lavorare? |
|  | SUPPORT INFORMATION | MATERIALE INFORMATIVO |
| 46 | What information is available about my cancer and its treatment, e.g. books, videos, websites, etc? | Che informazioni esistono relativamente al mio tumore e al suo trattamento (libri, video, siti internet, ecc.)? |
| 47 | Are there any complementary therapies that you believe may be helpful or that are known to be bad for me? | Esistono terapie alternative che lei ritiene potrebbero essere utili o dannose in casi come il mio? |
| 48 | Is there someone I can talk to who has been through this treatment? | E’ possibile parlare con qualcuno che si è sottoposto a questa terapia? |
| 49 | Are there services/support groups that can help me and my family deal with this illness? | Esistono servizi-gruppi di sostegno che potrebbero aiutare me e i miei familiari ad affrontare questa malattia? |
